# Analysis of the Interaction Network of Hub miRNAs-Hub Genes, Being Involved in Idiopathic Pulmonary Fibers and Its Emerging Role in Non-small Cell Lung Cancer

**DOI:** 10.3389/fgene.2020.00302

**Published:** 2020-04-02

**Authors:** Dong Hu Yu, Xiao-Lan Ruan, Jing-Yu Huang, Xiao-Ping Liu, Hao-Li Ma, Chen Chen, Wei-Dong Hu, Sheng Li

**Affiliations:** ^1^Department of Biological Repositories, Zhongnan Hospital, Wuhan University, Wuhan, China; ^2^Department of Hematology, Renmin Hospital, Wuhan University, Wuhan, China; ^3^Department of Thoracic Surgery, Zhongnan Hospital, Wuhan University, Wuhan, China; ^4^Human Genetics Resource Preservation Center, Wuhan University, Wuhan, China

**Keywords:** idiopathic pulmonary fibers, non-small cell lung cancer, weighted gene co-expression network analysis, hub genes, hub miRNAs, interaction network

## Abstract

Idiopathic pulmonary fibrosis (IPF) is a fibrotic interstitial lung disease with lesions confined to the lungs. To identify meaningful microRNA (miRNA) and gene modules related to the IPF progression, GSE32537 (RNA-sequencing data) and GSE32538 (miRNA-sequencing data) were downloaded and processed, and then weighted gene co-expression network analysis (WGCNA) was applied to construct gene co-expression networks and miRNA co-expression networks. GSE10667, GSE70866, and GSE27430 were used to make a reasonable validation for the results and evaluate the clinical significance of the genes and the miRNAs. Six hub genes (COL3A1, COL1A2, OGN, COL15A1, ASPN, and MXRA5) and seven hub miRNAs (hsa-let-7b-5p, hsa-miR-26a-5p, hsa-miR-25-3p, hsa-miR-29c-3p, hsa-let-7c-5p, hsa-miR-29b-3p, and hsa-miR-26b-5p) were clarified and validated. Meanwhile, iteration network of hub miRNAs-hub genes was constructed, and the emerging role of the network being involved in non-small cell lung cancer (NSCLC) was also analyzed by several webtools. The expression levels of hub genes were different between normal lung tissues and NSCLC tissues. Six genes (COL3A1, COL1A2, OGN, COL15A1, ASPN, and MXRA5) and three miRNAs (hsa-miR-29c-3p, hsa-let-7c-5p, and hsa-miR-29b-3p) were related to the survival time of lung adenocarcinoma (LUAD). The interaction network of hub miRNAs-hub genes might provide common mechanisms involving in IPF and NSCLC. More importantly, useful clues were provided for clinical treatment of both diseases based on novel molecular advances.

## Introduction

Idiopathic pulmonary fibrosis (IPF) is a chronic phlogistic interstitial lung disease with excessive tissue scarring and loss of function, and most patients with IPF would die of organ failure eventually (Datta et al., 2011; Lehtonen et al., 2016). To assess disease progression for the patients with IPF, the scores of St. George’s Respiratory Questionnaire (SGRQ) are usually used, which have a strong correlation with lung function significantly (Swigris et al., 2014, 2018; Lawrence et al., 2017). Besides, non-small cell lung cancer (NSCLC), which can mainly be categorized into lung adenocarcinoma (LUAD) and lung squamous cell carcinoma (LUSC), is commonly altering the course and mortality of IPF (Ballester et al., 2019). IPF and NSCLC are coexistent and affect each other, and majority of studies have shown that LUSC is the most frequent type of NSCLC in IPF patients, while LUAD is the second most frequent (Lee et al., 2014; Tomassetti et al., 2015; Kato et al., 2018). Studies have shown that the risk of NSCLC is higher in IPF patients, and it was reported that the cumulative prevalence of NSCLC is increased from IPF diagnosis (Kinoshita and Goto, 2019). Recent Studies indicated that the occurrence of IPF and NSCLC share the same genetic mutations and abnormal activation of signal pathways, suggesting potential molecular mechanisms between IPF and NSCLC, and there is speculation IPF could lead to cancer (Han et al., 2019; Kinoshita and Goto, 2019). IPF, which has a poor prognosis and a course that is unpredictable, thus needs for a more complete understanding of its mechanisms, and further research for IPF-NSCLC pathogenesis is also urgently needed.

MicroRNA (miRNA) is a class of gene regulator, and it can repress the expression of target genes by binding to the mRNAs (Taganov et al., 2007). In recent years, increasing evidences have revealed that multiple miRNAs can play as potential biomarkers for the prediction of IPF, including miR-92a (Berschneider et al., 2014), miR-let-7d (Huleihel et al., 2014), and miR-98 (Gao et al., 2014). However, studies of single miRNA cannot meet the requirement for exploration of IPF progression. miRNAs–mRNAs constitute networks, which are involved in many important cellular pathways, are badly needed to clarify exact mechanisms.

Though Fan has reported differently expressed genes and differently expressed miRNAs between normal tissue and IPF tissues (Fan et al., 2017), the relationships between hub genes and important clinical traits, hub miRNAs, and important clinical traits had not been rigorously studied. The weighted gene co-expression network analysis (WGCNA), which provides an effective way to explore the mechanisms behind certain traits, can solve this problem elegantly (Langfelder and Horvath, 2008). To fulfill these gaps, gene co-expression networks and miRNA co-expression networks were constructed by WGCNA to identify the gene and miRNA modules related to the scores of SGRQ in IPF, and the relationships between genes and miRNAs were predicted to construct miRNA–gene network, which would provide more information about the mechanisms of IPF progression, even IPF-NSCLC pathogenesis.

## Materials and Methods

### Data Collection and Processing

**FIGURE 1:**
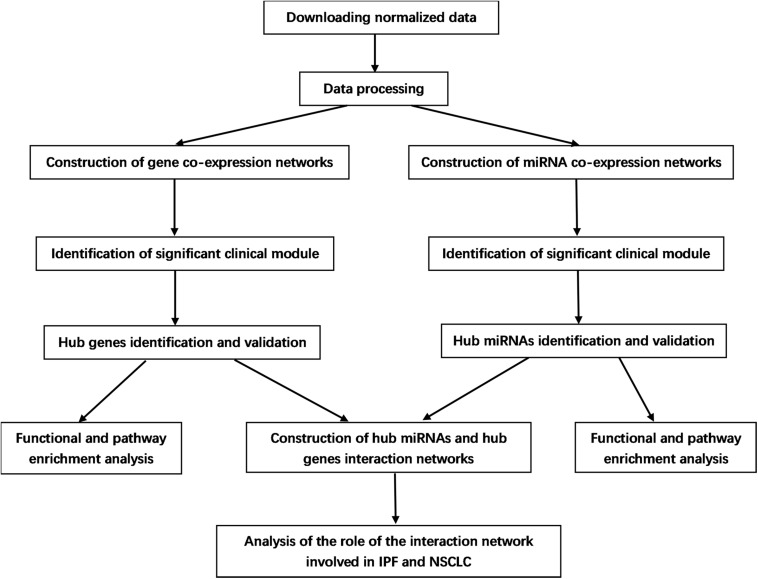
Flow chart of data preparation, processing, analysis, and validation.

A brief workflow for this study is indicated in Figure 1. Selection criteria on the Gene Expression Omnibus (GEO) database^1^ are: (1) The datasets contain miRNA expression profiles and gene expression profiles; (2) there are normal group (normal tissue samples) and IPF group (IPF tissue samples) in the datasets; and (3) the number of samples in each group is more than 10. miRNA expression profiles (GSE32538 and GSE27430) and gene expression profiles (GSE32537, GSE10667, and GSE70866) related to IPF were downloaded from GEO database. All datasets were normalized with quantile normalization. The data quality was evaluated, and boxplot was used to compare before and after being standardized. The details of these datasets are listed in Supplementary Table S1. Among them, GSE32537 and GSE32538 were used to identify hub genes and hub miRNAs by WGCNA separately. After doing analysis of variance for GSE32537, we chose the top 25% most variant genes (2987 genes) for constructing networks, while we did not to do pretreatment for GSE32538 due to the small number of miRNAs (1801 miRNAs).

### Construction of Co-expression Networks

Weighted gene co-expression network analysis was used to construct gene co-expression networks and miRNA co-expression networks (Langfelder and Horvath, 2008). The processes for constructing gene co-expression networks and miRNA co-expression networks were similar. So, we took the construction of weighted gene co-expression networks as an example. First, a matrix of similarity was constructed by calculating the correlations of the processed genes. Second, an appropriate power of β was chosen as the soft-thresholding parameter to construct a scale-free network. Third, the adjacency was transformed into a topological overlap matrix (TOM) by using TOM similarity, and the corresponding dissimilarity (1-TOM) was figured and the dissimilarity of module eigengenes (MEs) was estimated. Fourth, the genes with similar expression levels were categorized into the same module by DynamicTreeCut algorithm.

### Identification of Clinically Significant Modules

The clinical trait that we concerned was the scores of SGRQ in IPF patients and key modules needed to be found in two networks separately. Above all, we worked out the relationship between clinical phenotype and MEs. MEs were deemed to represent the expression levels of all genes or miRNAs in the related module. In addition, mediated *p*-value of each gene or miRNA was calculated and then we worked out gene significance or miRNA significance (GS = lg P). Finally, we selected the most clinically significant module according to module significance (MS), which was the average GS of genes or miRNAs involved in the related module.

### Functional and Pathway Enrichment Analysis

The Database for Annotation, Visualization and Integrate Discovery5 (DAVID)^2^ is a database for several kinds of functional annotation (Huang et al., 2009). With the help of DAVID, we identified biological meaning of the genes in a given module according to false discovery rate (FDR) < 0.05. GO includes three terms: biological process (BP), cellular component (CC), and molecular function (MF). Besides, GO (BP, CC, MF) and KEGG enrichment analyses for the miRNAs in the selected module were conducted using mirPath v.3, an online tool for miRNA pathway analysis (Vlachos et al., 2015).

### Identification and Validation of Hub Genes and Hub miRNAs in IPF

The connectivity of module can be measured by absolute value of the Pearson’s correlation. Besides, the relationship between clinical trait and genes can be measured by absolute value of the Pearson’s correlation. The genes that have high connectivity with module and selected phenotype were selected as candidate genes in hub module (cor. geneModuleMembership > 0.8 and cor. geneTraitSignificance > 0.2). Then the protein/gene interactions for candidate genes were analyzed using STRING (Szklarczyk et al., 2019) and the genes connected with more than five nodes in PPI network were selected as hub genes for further study. As for selecting hub miRNAs, two web tools, microT-CDS^3^ and TargetScan^4^, were employed to predict candidate miRNAs for hub genes (Paraskevopoulou et al., 2013; Agarwal et al., 2015), and the score of microT-CDS > 0.9 and context + + score of TargetScan > 0.4 were selected as threshold. Then the common candidate miRNAs in hub module and prediction by microT-CDS and TargetScan were defined as real hub miRNAs. To verify our results, GSE10667 (including 15 normal lung tissues and 31 IPF tissues) and GSE70866 (including 20 normal lung tissues and 110 IPF tissues), were used to validate the different expression levels of hub genes between normal tissue and IPF tissues with two-tailed student’s *t*-tests, separately.

### Gene Set Enrichment Analysis (GSEA) and Guilt of Association for Hub Genes

Gene set enrichment analysis (GSEA) analysis was performed for hub genes in GSE32537 (Subramanian et al., 2005). In GSE32537, according to the median expression of this hub gene, 119 cases were classified into high expression group and low expression group (high group, *n* = 60; low group, *n* = 59). | ES| > 0.5, nominal *P* < 0.05, and FDR ≥ 25% were chosen as the cut-off criteria. Besides, Spearman correlation analysis was performed to explore pair-wise gene expression correlation for hub genes in GSE10667. We calculated correlation coefficient absolute values, and the top 300 genes of each hub gene were selected for functional enrichment analysis. Based on the results, the potential functions of each hub gene were predicted, and the method thus bore the name of “guilt of association.”

### Construction of Hub miRNA and Hub Gene Interaction Network

According to the score of microT-CDS and the context + + score of TargetScan, miRNA–gene interaction network was constructed in Cytoscape (Shannon et al., 2003). And the interaction between genes was also demonstrated from STRING. Furthermore, text mining of hub genes and hub miRNAs was performed using GenCLip 2.0^5^. GenCLip 2.0 is an online text-mining server, which can provide the analysis of gene and miRNA functions with free terms generated by literature mining (Wang et al., 2014).

### Analysis of the Role of the Interaction Network Involved in IPF and NSCLC

To further understand the role of hub genes and hub miRNAs in clinical practice, we selected two data sets (GSE70866 and GSE27430) with clearer clinical information to do clinicopathological correlation analysis separately. From GSE70866, 110 samples with IPF were used to determine the association between age and hub genes expression levels, between gender and hub genes expression levels by Pearson Chi-square test. From GSE27430, 13 samples with IPF were used to determine the association between age and hub miRNAs expression levels, gender, and hub miRNAs expression levels with Fisher test due to small sample size. *P*-value < 0.05 was considered as statistical significance. In addition, to explore the role of the interaction network in NSCLC (mainly including LUAD and LUSC), UALCAN^6^ was used to explore the different expression levels of hub genes between normal tissues and cancer tissues (including LUAD and LUSC), separately. UALCAN is a useful online tool for analyzing cancer transcriptome data, which is based on public cancer transcriptome data (TCGA and MET500 transcriptome sequencing) (Chandrashekar et al., 2017). Moreover, we evaluate the relationship between the expression levels of hub genes and the prognosis of LUAD and LUSC, the expression levels of hub miRNAs and the prognosis of LUAD and LUSC. Kaplan Meier Plotter^7^, including the gene expression data and survival information of GEO and TCGA repositories, was used to explore the relationship between the expression levels of hub genes and the survival time of LUAD and LUSC (Gyoerffy et al., 2014). Besides, OncoLnc^8^, containing survival data from 21 cancer studies performed by TCGA and giving users the ability to create publication-quality Kaplan–Meier plots, was used to explore the relationship between the expression levels of hub miRNAs and the survival time of LUAD and LUSC (Anaya, 2016).

## Results

### Weighted Co-expression Networks Construction and Key Modules Identification

**FIGURE 2:**
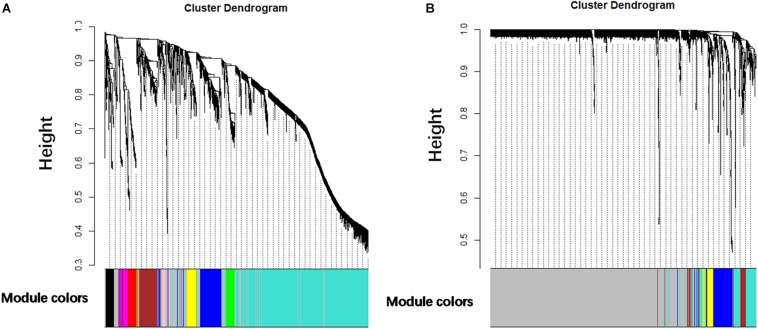
Clustering dendrograms. **(A)** Clustering dendrograms of genes based on a dissimilarity measure (1-TOM). **(B)** Clustering dendrograms of miRNAs based on a dissimilarity measure (1-TOM).

**FIGURE 3:**
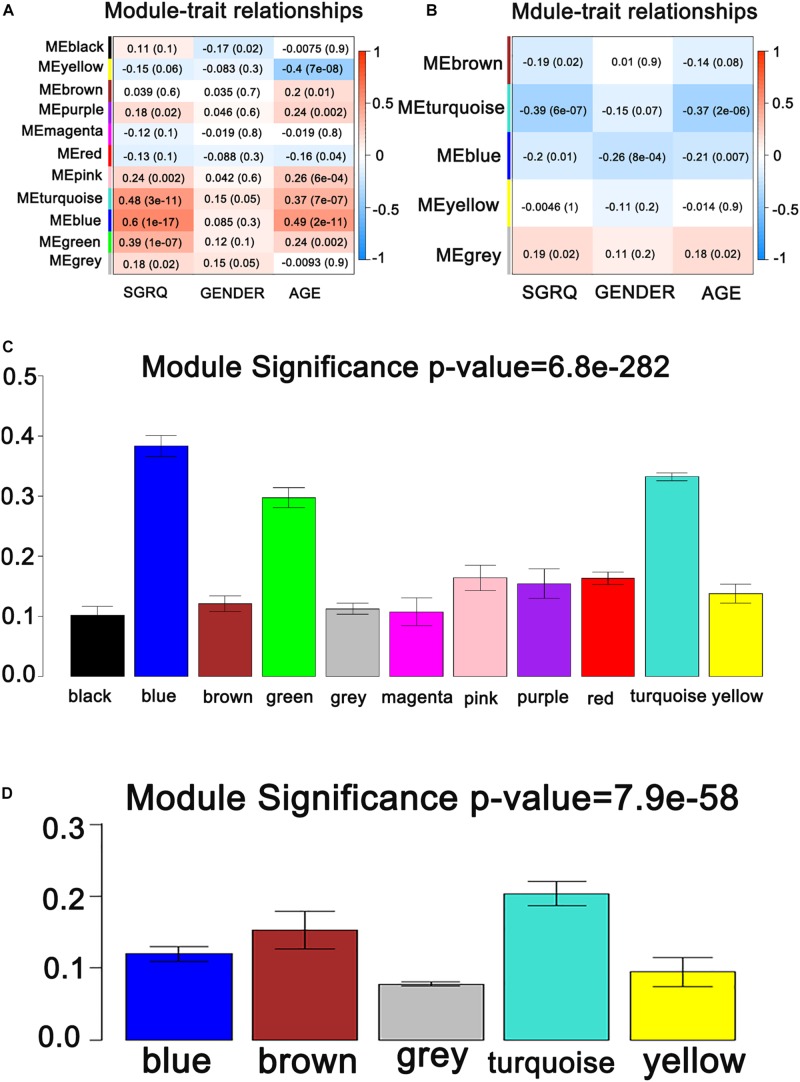
Identification of modules associated with the clinical traits of IPF. **(A)** Heatmap of the correlation between co-expressed gene module eigengenes and clinical traits of IPF. **(B)** Heatmap of the correlation between co-expressed miRNA module eigengenes and clinical traits of IPF. **(C)** Distribution of average gene significance and errors in the modules associated with the scores of SGRQ. **(D)** Distribution of average miRNA significance and errors in the modules associated with the scores of SGRQ in IPF.

It is found that the median of miRNA/gene expression value of each sample is approximately equal (Supplementary Figure S1), and the results indicated that the processed datasets can be used for further analysis. With the method of average linkage hierarchical clustering, the samples of both data sets (GSE32537 and GSE32538) are well clustered separately. The clustering dendrograms of the genes of GSE32537 are generated in Figure 2A, while miRNAs of GSE32538 are shown in Figure 2B. By “WGCNA” package in R, the genes and the miRNAs which had similar expression levels were divided into modules to construct co-expression networks. Power of β = 3 (scale free *R*^2^ = 0.92) was selected as the soft-thresholding parameter for gene co-expression networks (Supplementary Figure S2), and power of β = 5 (scale free *R*^2^ = 0.89) was selected for miRNA co-expression networks (Supplementary Figure S3). In gene co-expression networks, 11 modules were identified and blue module (GS = 0.38, *p*-value = 6.8e-282) showed the highest correlation with the scores of SGRQ. In miRNA co-expression networks, five modules were identified and turquoise module (GS = 0.20, *p*-value = 7.9e-58) showed the highest correlation with the scores of SGRQ (Figure 3). There are 285 genes in blue module and 163 miRNAs in turquoise module. Blue module (G blue) and turquoise module (M turquoise) were picked for following analysis as the clinically significant module.

### Pathway Enrichment Analysis of Genes and miRNAs in Hub Modules

**FIGURE 4:**
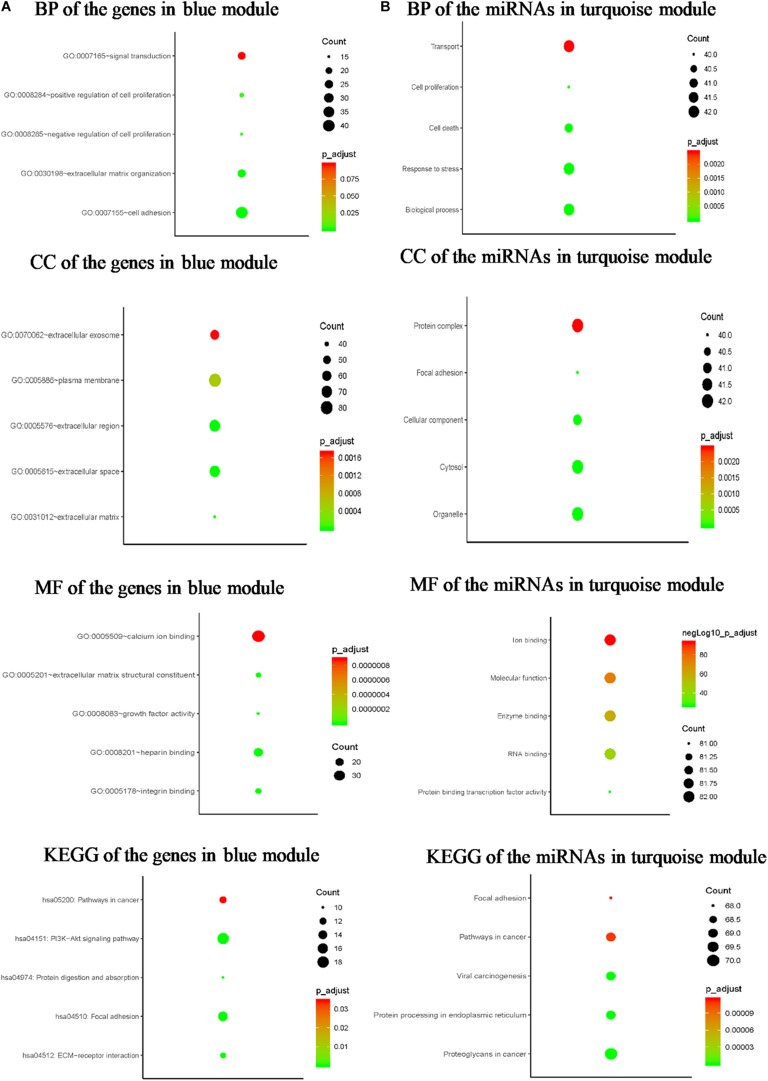
Bioinformatics analysis of the genes in blue module and the miRNAs in turquoise module. **(A)** GO analysis and KEGG pathway enrichment of the genes in blue module. **(B)** GO analysis and KEGG pathway enrichment of the miRNAs in turquoise module.

To explore the biological functions of the G blue, the genes were categorized into BP, CC, and MF. The outcome of GO and KEGG enrichment of the genes in blue module was shown in Figure 4A. The genes in BP were generally enriched in cell adhesion, extracellular matrix organization, signal transduction, positive regulation of cell proliferation, and negative regulation of cell proliferation; the genes in CC were mainly focused on plasma membrane, extracellular region, extracellular space, extracellular exosome, and extracellular matrix; the genes in MF were significantly focused on calcium ion binding, heparin binding, integrin binding, extracellular matrix structural constituent, and growth factor activity. The top five significantly enriched pathways in blue module were PI3K-Akt signaling pathway, focal adhesion, pathways in cancer, ECM–receptor interaction, and protein digestion and absorption. Top enriched GO terms for the miRNAs in turquoise module were: BP, transport, response to stress, cell death, and cell proliferation in BP; organelle, protein complex, cytosol, CC, and focal adhesion in CC; ion binding, MF, enzyme binding, RNA binding, and protein binding transcription factor activity in MF. The pathway analysis was also performed for the miRNAs in turquoise module. The top five significantly enriched pathways were proteoglycans in cancer, protein processing in endoplasmic reticulum, viral carcinogenesis, pathways in cancer, and focal adhesion (Figure 4B).

### Identification and Validation of Hub Genes and miRNAs in IPF

**FIGURE 5:**
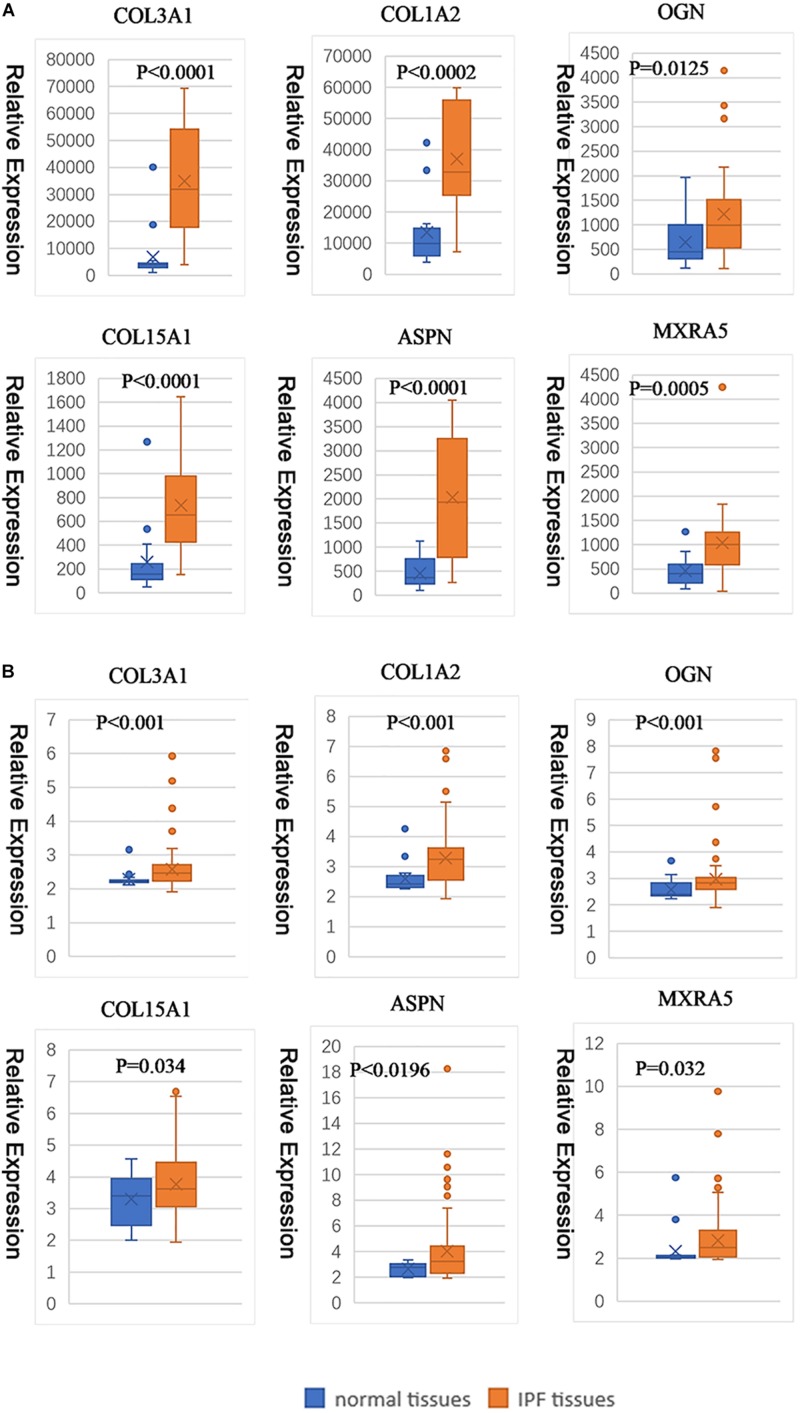
Hub gene expression levels between normal tissue and IPF tissue (based on GSE10667 and GSE70866). The gene expression levels of COL3A1, COL1A2, OGN, COL15A1, ASPN, and MXRA5 in GSE10667 **(A)**. The gene expression levels of COL3A1, COL1A2, OGN, COL15A1, ASPN, and MXRA5 in GSE70866 **(B)**.

Under the threshold of | MM| > 0.8 and | GS| > 0.2, 58 genes in blue module were considered as candidate genes. Then the relationship between candidate genes was identified from STRING (Supplementary Figure S4), and we calculated the connectivity degree of each node in PPI. The nodes with degrees ≧5 were COL3A1, COL1A2, OGN, COL15A1, ASPN, and MXRA5, which were considered as real hub gens because it interacted with more proteins. Based on the prediction of microT-CDS and TargetScan, seven hub miRNAs (hsa-let-7b-5p, hsa-miR-26a-5p, hsa-miR-25-3p, hsa-miR-29c-3p, hsa-let-7c-5p, hsa-miR-29b-3p, and hsa-miR-26b-5p) were identified in turquoise module. In the blue module, COL3A1 and COL1A2 were the most central genes with the degrees of 13, and they are involved in the process of other genes regulating cell metabolism. As for the miRNAs, hsa-let-7b-5p was considered as key miRNA with the highest MM (MM = 0.915). The corresponding MM and GS of hub genes and hub miRNAs are shown in Table 1. From the results of two-tailed student’s *t*-tests for GSE10667 and GSE70866, the expression levels of all hub genes (COL3A1, COL1A2, OGN, COL15A1, ASPN, and MXRA5) were significantly higher in IPF tissues (Figure 5). And the ROC curve analysis for GSE10067 indicated that the hub genes exhibited excellent diagnostic efficiency for normal tissues and IPF tissues (Supplementary Figure S5).

**TABLE 1:** The hub genes and hub miRNAs as well as the corresponding MM and GS.

	**Symbol**	**Degrees in PPI**	**MM**	**GS**
Hub	COL3A1	13	0.812933	0.582487
genes	COL1A2	13	0.821623	0.555745
	OGN	6	0.862299	0.475489
	COL15A1	5	0.860161	0.600621
	ASPN	5	0.854841	0.642866
	MXRA5	5	0.805921	0.592231
Hub	hsa-let-7b-5p	−	0.915297	–0.35161
miRNAs	hsa-miR-26a-5p	−	0.825955	–0.44743
	hsa-miR-25-3p	−	0.793815	–0.31258
	hsa-miR-29c-3p	−	0.676672	–0.25468
	hsa-let-7c-5p	−	0.660136	–0.1454
	hsa-miR-29b-3p	−	0.622602	–0.19913
	hsa-miR-26b-5p		0.577243	–0.13157

*miRNAs: microRNAs. PPI: protein/gene interactions. MM: cor.geneModuleMembership. GS: cor.geneTraitSignificance.*

### GSEA and Guilt of Association

Gene set enrichment analysis was performed to identify the lurking mechanisms related to IPF progression of six hub genes. As shown in Supplementary Table S2, IPF samples in COL3A1 high expression group were most significantly enriched in cellular adhesion molecules; IPF samples in COL1A2, OGN, COL15A1, ASPN, and MXRA5 high expression groups were most significantly enriched in ECM receptor interaction (Supplementary Tables S2–S7). Based on the analysis of guilt of association, we identified that the hub genes were essential for extracellular environment and ossification, and they mainly played important roles in extracellular structure organization, extracellular matrix organization, and skeletal system development (Supplementary Figure S6).

### Construction of Hub miRNA and Hub Gene Interaction Network

**FIGURE 6:**
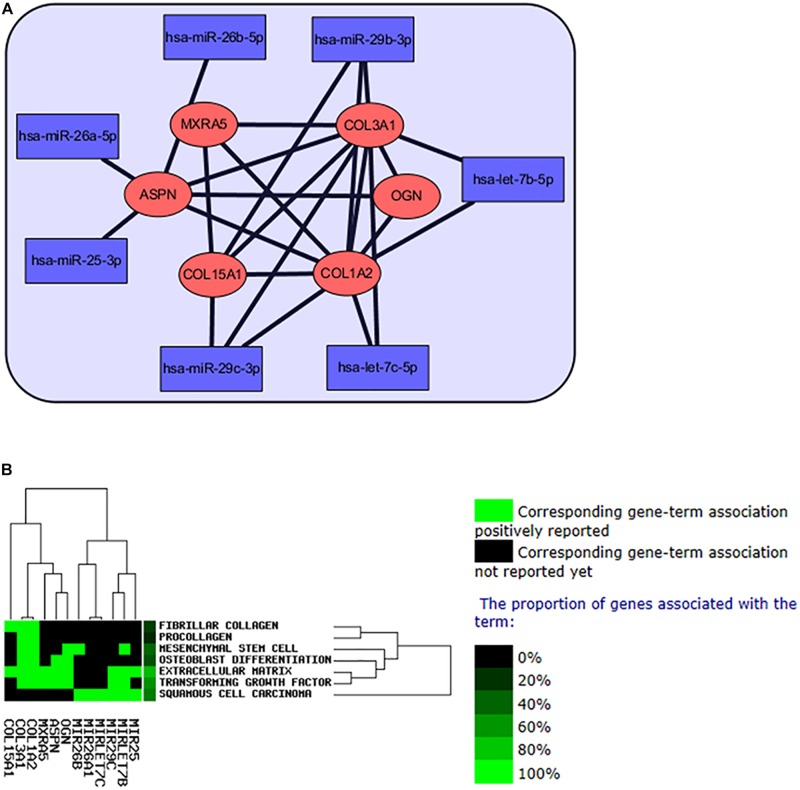
The interaction network of hub miRNAs and hub genes. **(A)** The network of regulation of hub miRNAs and hub genes in IPF. **(B)** Text mining of the hub genes and hub miRNAs from GenCLip 2.0 software.

The hub genes and hub miRNAs interactions were predicted by microT-CDS and Targetscan (Table 2), and the hub genes and hub miRNAs interaction network was shown in Figure 6A. Six genes (COL3A1, COL1A2, OGN, COL15A1, ASPN, and MXRA5) and seven miRNAs (hsa-let-7b-5p, hsa-miR-26a-5p, hsa-miR-25-3p, hsa-miR-29c-3p, hsa-let-7c-5p, hsa-miR-29b-3p, and hsa-miR-26b-5p) were involved in this interaction network. Besides, the occurrence frequency of terms of corresponding literature was demonstrated from GenCLip 2.0, including extracellular matrix, transforming growth factor, squamous cell carcinoma, mesenchymal stem cell, fibrillar collagen, procollagen, and osteoblast differentiation (Figure 6B).

**TABLE 2:** The prediction of the interaction of hub genes and hub miRNAs by microT-CDS and Targetscan.

**miRNA**	**Target**	**Score of**	**Context + + score**
	**gene**	**microT-CDS**	**of TargetScan**
hsa-let-7b-5p	COL3A1	0.99	–0.47
hsa-let-7b-5p	COL1A2	0.99	–0.5
hsa-miR-26a-5p	ASPN	0.99	–0.41
hsa-miR-25-3p	ASPN	0.93	–0.44
hsa-miR-29c-3p	COL3A1	0.99	–0.87
hsa-miR-29c-3p	COL1A2	0.99	–0.61
hsa-miR-29c-3p	COL15A1	0.99	–0.5
hsa-let-7c-5p	COL3A1	0.99	–0.47
hsa-let-7c-5p	COL1A2	0.99	–0.5
hsa-miR-29b-3p	COL3A1	0.99	–0.87
hsa-miR-29b-3p	COL1A2	0.99	–0.61
hsa-miR-29b-3p	COL15A1	0.99	–0.52
hsa-miR-26b-5p	ASPN	0.98	–0.4

*miRNA: microRNA.*

### Analysis of Hub Genes–Hub miRNAs Interaction Network in IPF and NSCLC

**FIGURE 7:**
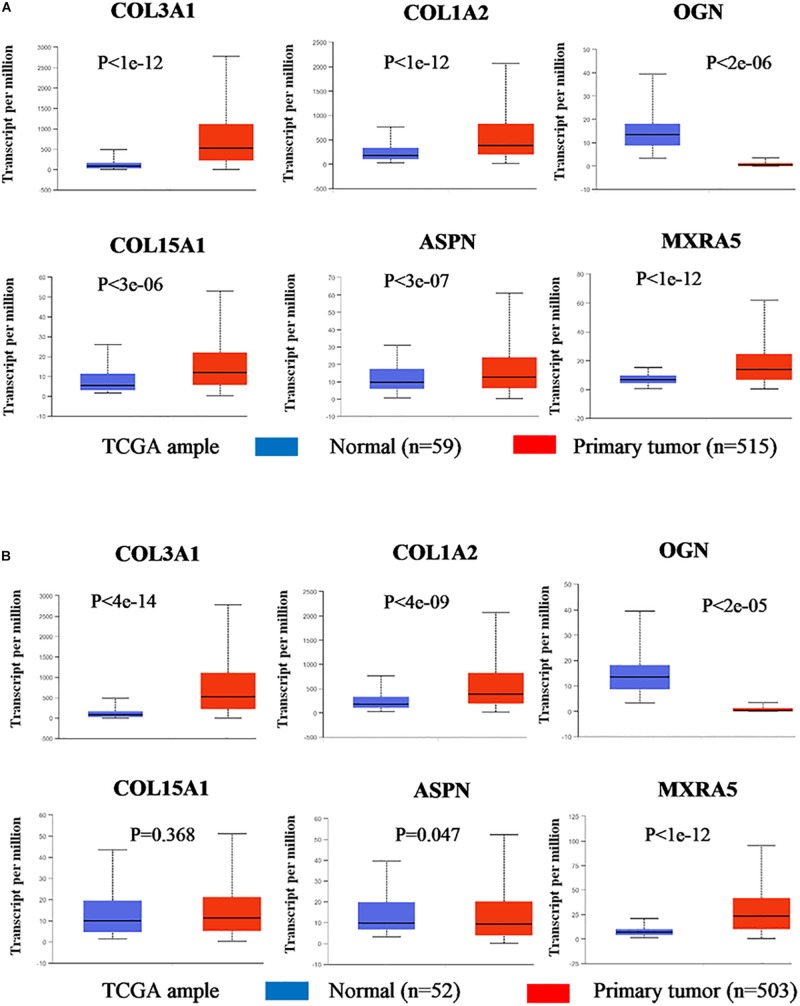
Gene expression levels between normal lung and tumor samples (based on TCGA data in UALCAN). **(A)** The gene expression levels of COL3A1, COL1A2, OGN, COL15A1, ASPN, and MXRA5 between normal lung tissues and LUAD tissues. **(B)** The gene expression levels of COL3A1, COL1A2, OGN, COL15A1, ASPN, and MXRA5 between normal lung tissues and LUSC tissues.

**FIGURE 8:**
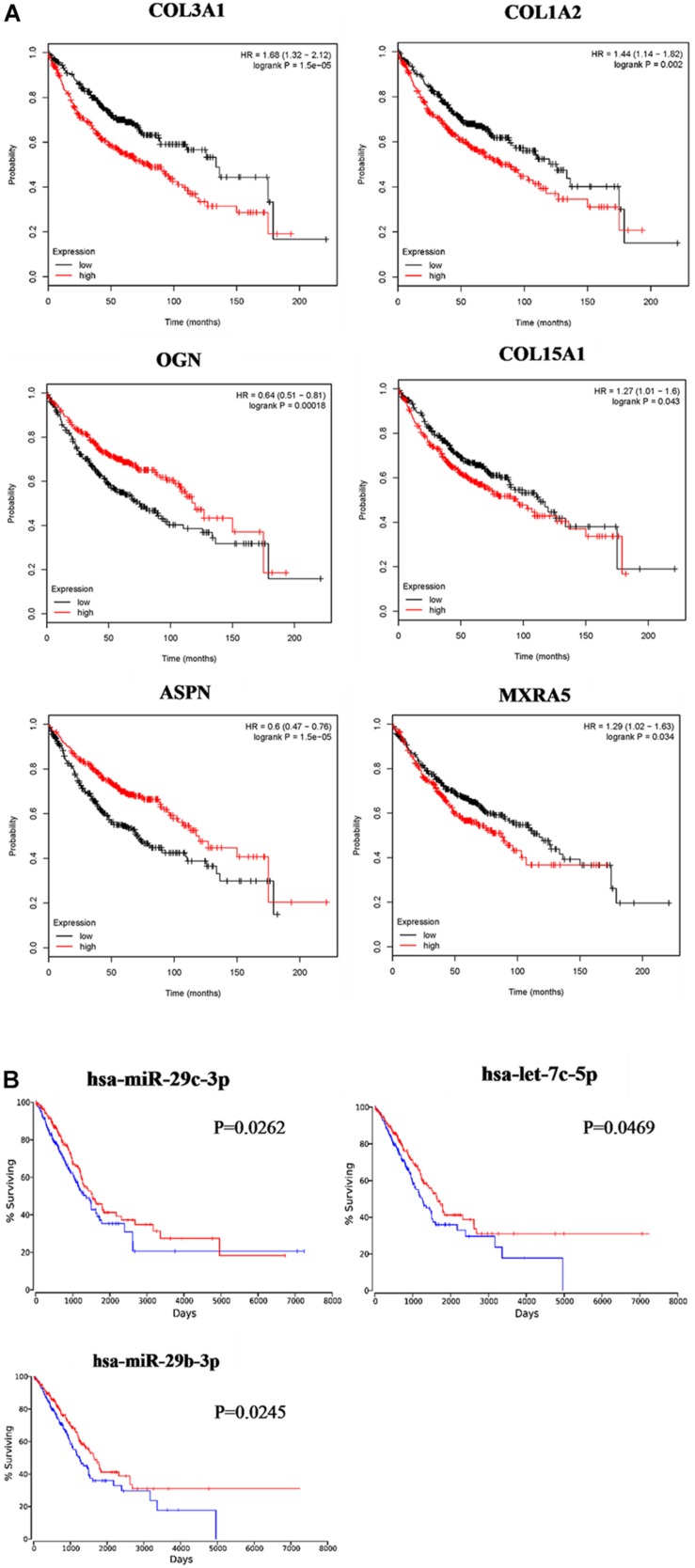
Survival analysis of the association between the expression levels of hub genes and hub miRNAs in LUAD. COL3A1, COL1A2, OGN, COL15A1, ASPN, and MXRA5 were identified to be related to the overall survival of LUAD from Kaplan Meier Plotter **(A)**. hsa-miR-29c-3p, hsa-let-7c-5p, and hsa-miR-29b-3p were identified to be related to the overall survival of LUAD from OncoLnc **(B)**.

Based on the results of clinicopathological correlation analysis, there were no statistical differences in age distribution and gender distribution between these high-expression and low-expression groups of hub genes. And we also did not find any substantial differences in age distribution and gender distribution between these high-expression and low-expression groups of hub miRNAs. More details are listed in Supplementary Table S8. Furthermore, some databases were used to explore the role of the interaction network in NSCLC (LUAD and LUSC). The levels of the six genes (COL3A1, COL1A2, OGN, COL15A1, ASPN, and MXRA5) expression were significantly different between normal samples and LUAD samples from UALCAN (Figure 7A). COL3A1, COL1A2, COL15A1, ASPN, and MXRA5 were higher expressed in tumor samples, while OGN was lower expressed. In LUSC tissues, the levels of COL3A1, COL1A2, OGN, ASPN, and MXRA5 expressions were significantly different from normal lung tissues, and there is no difference of COL15A1 between normal tissues and LUSC tissues (Figure 7B). For the relationship between hub genes expression levels and the prognosis of NSCLC from Kaplan Meier Plotter, COL3A1, COL1A2, OGN, COL15A1, ASPN, and MXRA5 were associated with the overall survival of LUAD (Figure 8A), but the expression levels of these genes did not affect overall survival of LUSC patients. Besides, hsa-miR-29c-3p, hsa-let-7c-5p, hsa-miR-29b-3p were identified to be related to the overall survival of LUAD from OncoLnc (Figure 8B).

## Disscusion

Idiopathic pulmonary fibrosis is a medically incurable disease with complicated clinical manifestations. Nowadays, only two medicines, nintedanib and pirfenidone, are approved for the treatment to slow down the progression of IPF (Lehtonen et al., 2016; Maher et al., 2017; Drakopanagiotakis et al., 2018). In order to identify a meaningful biomarker, a part of previous studies had focused too much on single miRNA or gene (Mizuno et al., 2017), and this cannot meet the requirement for exploration of molecular mechanisms in IPF progression. Though another part of previous studies had reported differently expressed genes and differently expressed miRNAs between normal tissue and IPF tissues to further explore the molecular mechanisms, the relationships between hubs and important clinical traits had not been rigorously studied, which would make clinically significance few. Besides, there are some previous studies focusing preclinical models by aberrant gene expression; though these modules are useful for clinical application, it did not make much sense in exploration of pathogenesis in IPF and NSCLC. It is a pity that the research on molecular mechanisms of IPF affecting NSCLC occurrence and prognosis was little, especially in bioinformatics. To fulfill these gaps, the interaction network of hub miRNAs-hub genes was studied on this research, and WGCNA was used to identify IPF gene and miRNA modules for the first time. More importantly, it was the first time to explore the common mechanisms and molecular targets between IPF and NSCLC in bioinformatics, which would provide more information about that IPF causing NSCLC and poor NSCLC prognosis, and this more attention is to be called on IPF-NSCLC patients. Two modules were found, including one gene module (blue module) and one miRNA module (turquoise module), were significantly related to the scores of SGRQ. We identified six hub genes and seven hub miRNAs, and the hub miRNAs–hub genes interaction network was constructed. In GenCLip 2.0, the BPs (extracellular matrix, transforming growth factor, squamous cell carcinoma, mesenchymal stem cell, etc.) were considered to be significantly related to IPF and NSCLC.

Focal adhesion was considered as a key pathway shared by blue module and turquoise module, and many gens/proteins have been considered to be involved in the progression of IPF through disordering focal adhesion (Gimenez et al., 2017; Kathiriya et al., 2017; Molina-Molina et al., 2018). For example, it has been reported that decreased expression of collagen VI, an important kind of protein of ECM, would upregulate the focal adhesion (Knueppel et al., 2018). For example, COL1A2, which is a subtype of Type I collagen (Fang et al., 2019), is implicated in the induction of epithelial–mesenchymal transition in many fibroblasts (Cheng et al., 2017). Type I collagen could induce the disruption of E−cadherin33 and SMADS to downregulate E−cadherin (Koenig et al., 2006). Of course, there are still potential pathways worth further study about hub genes in IPF. In present study, the hub miRNAs, except hsa-miR-25-3p (Min et al., 2016), were identified to be related to the progression of IPF for the first time, which would be novel diagnostic biomarkers of patients with IPF.

After analyzing and comparing the results of GSEA analysis and guilt of association, we found that ECM–receptor interaction is an important pathway shared by hub genes. Pulmonary extracellular matrix, which is a complex system composed of proteoglycans and glycosaminoglycans, is of importance in tissue’s homeostasis and repair. Previous studies have revealed that ECM protein expression plays an important role in the fibrotic process in IPF lungs (Vicens-Zygmunt et al., 2015). Excessive accumulation of ECM in the alveolar parenchyma and progressive scarring of lung tissue are major characteristics of IPF (Knudsen et al., 2017), and some studies have used this protein expression level as a criterion for evaluating treatment outcomes (Molina-Molina et al., 2018; Mullenbrock et al., 2018). Altogether, migration is strongly influenced by topology and composition of the ECM including integrin ligands, and the hub gens and hub miRNAs might play an important role in IPF progression with the change of ECM.

Evidence suggests that patients with NSCLC who develop IPF have worse outcomes than patients without IPF (Han et al., 2019). Clinical examples with both diseases are numerous, and they are difficult to treat. In the treatment of patients suffered IPF and NSCLC, physicians are reluctant to treat NSCLC because of the poor prognosis of IPF (Kinoshita and Goto, 2019). Therefore, the interaction network was analyzed between these two types of diseases, which would provide more information about that IPF causing NSCLC and poor NSCLC prognosis. Though cancer was not taken as the main research topic at first, with analysis continuing, we identified hub miRNAs and hub genes may participate in the progression of NSCLC. And the hub miRNAs–hub genes interaction network would help us understand the pathogenesis of IPF-NSCLC. For example, COL3A1 is highly expressed in both IPF and NSCLC tissues, so it is speculated that COL3A1 is a key molecule of cross-linking between IPF and NSCLC, and even a signal of IPF leading to NSCLC. MXRA5 is upregulated in IPF, and it is found that the higher the expression, the worse the prognosis of NSCLC. We speculated that MXRA5 is an important intermediate molecule of IPF leading to poor prognosis of NSCLC. Of course, these all need further experimental verification later, and some experiments need to be done to confirm the hub genes. We will further explore the hubs and its role in the progression of IPF-NSCLC by using more in-depth bioinformatic analyses and experimental methods in the future., In this study, OGN was identified to be related to the progression of IPF for the first time. Most interestingly, we found that OGN is highly expressed in IPF, but is lowly expressed in cancer tissues. And low expression levels of OGN would have an important impact on the prognosis of LUAD (Figure 8). Different signal pathways should be activated to regulate or influence OGN. Although many studies identified that the expression levels of OGN would alter in cancers, such as gastric cancer (Lee et al., 2003), colorectal cancer (Hu et al., 2018), and invasive ductal breast carcinoma (Roewer et al., 2011), functional data about how OGN participating in cancer pathology are not enough, and further studies are needed.

## Conclusion

It was the first time to construct miRNA–gene interaction network to explore the development of IPF and common pathways between IPF and NSCLC by WGCNA. We identified six hub genes (COL3A1, COL1A2, OGN, COL15A1, ASPN, and MXRA5) and seven hub miRNAs (hsa-let-7b-5p, hsa-miR-26a-5p, hsa-miR-25-3p, hsa-miR-29c-3p, hsa-let-7c-5p, hsa-miR-29b-3p, and hsa-miR-26b-5p), which might be diagnostic biomarkers for IPF. In the future, the pathogenic overlap of IPF and NSCLC may help us to clarify the common molecular mechanisms between both diseases, and may provide a potential treatment strategy for both diseases.

## Data Availability Statement

The data analyzed in this study can be found in the GEO database (http://www.ncbi.nlmNih.gov/geo), using accession numbers GSE32537, GSE10667, GSE70866, GSE32538, and GSE27430.

## Author Contributions

DY, X-LR, X-PL, and SL reviewed relevant literature and drafted the manuscript. DY, X-LR, J-YH, H-LM, CC, and W-DH conducted all statistical analyses. All authors read and approved the final manuscript.

## Conflict of Interest

The authors declare that the research was conducted in the absence of any commercial or financial relationships that could be construed as a potential conflict of interest.

## Footnotes

1 https://www.ncbi.nlm.nih.gov/geo/

2 https://david.ncifcrf.gov/

3 http://www.microrna.gr/microT-CDS/

4 http://www.targetscan.org/

5 http://ci.smu.edu.cn/

6 http://ualcan.path.uab.edu/

7 http://kmplot.com/analysis/

8 http://www.oncolnc.org/

## Supplementary Material

FIGURE S1 Standardization of gene expression. The data quality was evaluated, and boxplot was used to compare before and after being standardized.

FIGURE S2 Determination of soft-thresholding power in the weighted gene co-expression network analysis (WGCNA). **(a)** Analysis of the scale-free fit index for various soft-thresholding powers. **(b)** Analysis of the mean connectivity for various soft-thresholding powers. **(c)** Histogram of connectivity distribution when β = 3. **(d)** Checking the scale free topology when β = 3.

FIGURE S3 Determination of soft-thresholding power in the weighted miRNA co-expression network analysis. **(a)** Analysis of the scale-free fit index for various soft-thresholding powers. **(b)** Analysis of the mean connectivity for various soft-thresholding powers. **(c)** Histogram of connectivity distribution when β = 5. **(d)** Checking the scale free topology when β = 5.

FIGURE S4 Protein–protein interaction network of 58 candidate genes acquired from STRING 9.1.

FIGURE S5 ROC curve of COL3A1, COL1A2, OGN, COL15A1, ASPN, and MXRA5 in GSE10067.

FIGURE S6 Guilt of association for hub genes (COL3A1, COL1A2, OGN, COL15A1, ASPN, and MXRA5).

TABLE S1 Gene and miRNA expression microarray datasets related to IPF.

TABLE S2 Gene set enriched in lung samples with COL3A1 high expression.

TABLE S3 Gene set enriched in lung samples with COL1A2 high expression.

TABLE S4 Gene set enriched in lung samples with OGN high expression.

TABLE S5 Gene set enriched in lung samples with COL15A1 high expression.

TABLE S6 Gene set enriched in lung samples with ASPN high expression.

TABLE S7 Gene set enriched in lung samples with MXRA5 high expression.

TABLE S8 Clinicopathological correlation analysis for hub genes and hub miRNAs in IPF.
