# Supplementary figures and images for: Analysis of the Interaction Network of Hub miRNAs-Hub Genes, Being Involved in Idiopathic Pulmonary Fibers and Its Emerging Role in Non-small Cell Lung Cancer
Source: Front Genet. 2020 Apr 2;11:302. doi: 10.3389/fgene.2020.00302 (PMC7142269; doi:10.3389/fgene.2020.00302)

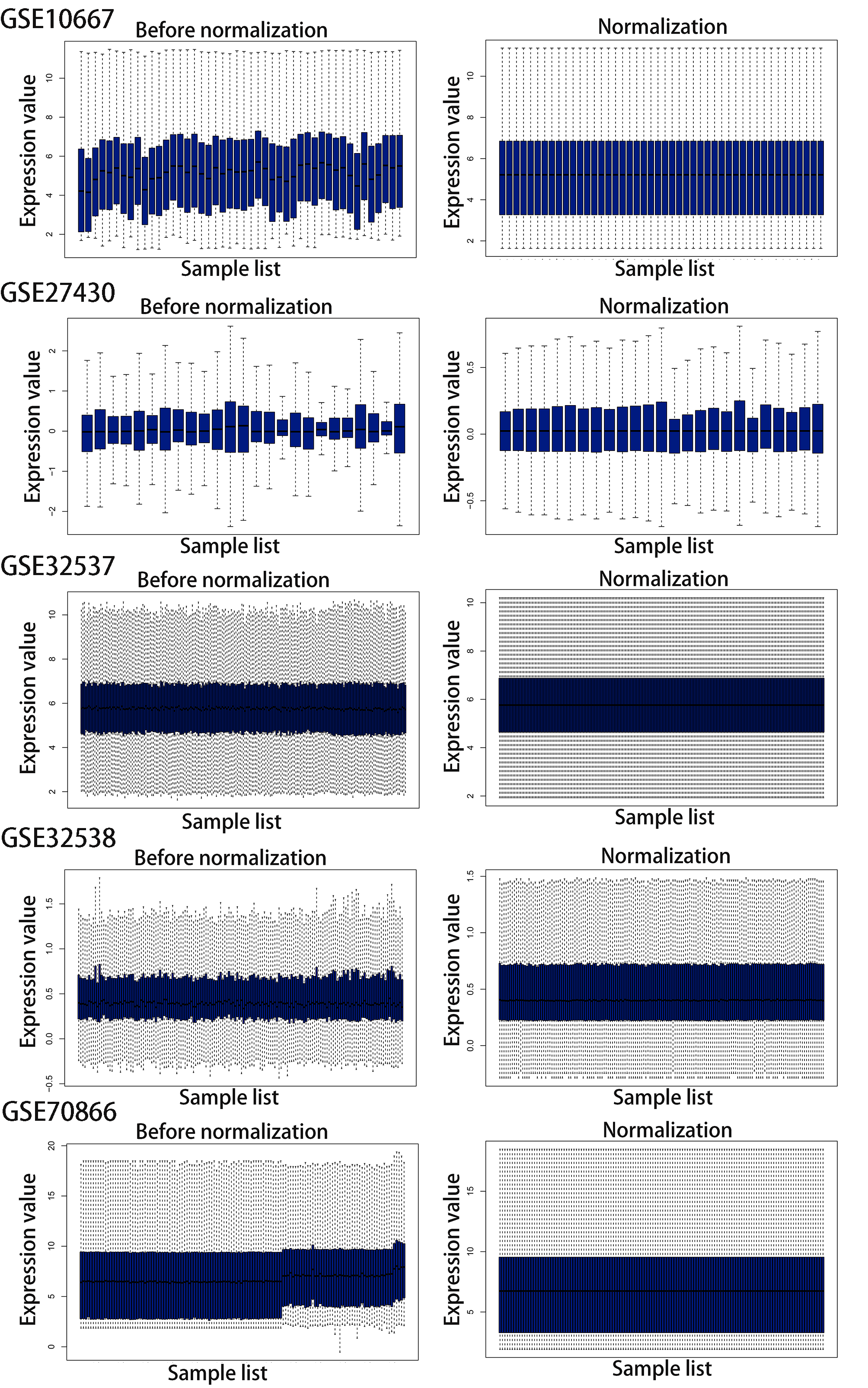

Supplement: FIGURE S1 — Standardization of gene expression. The data quality was evaluated, and boxplot was used to compare before and after being standardized. [file Image_1.TIF]

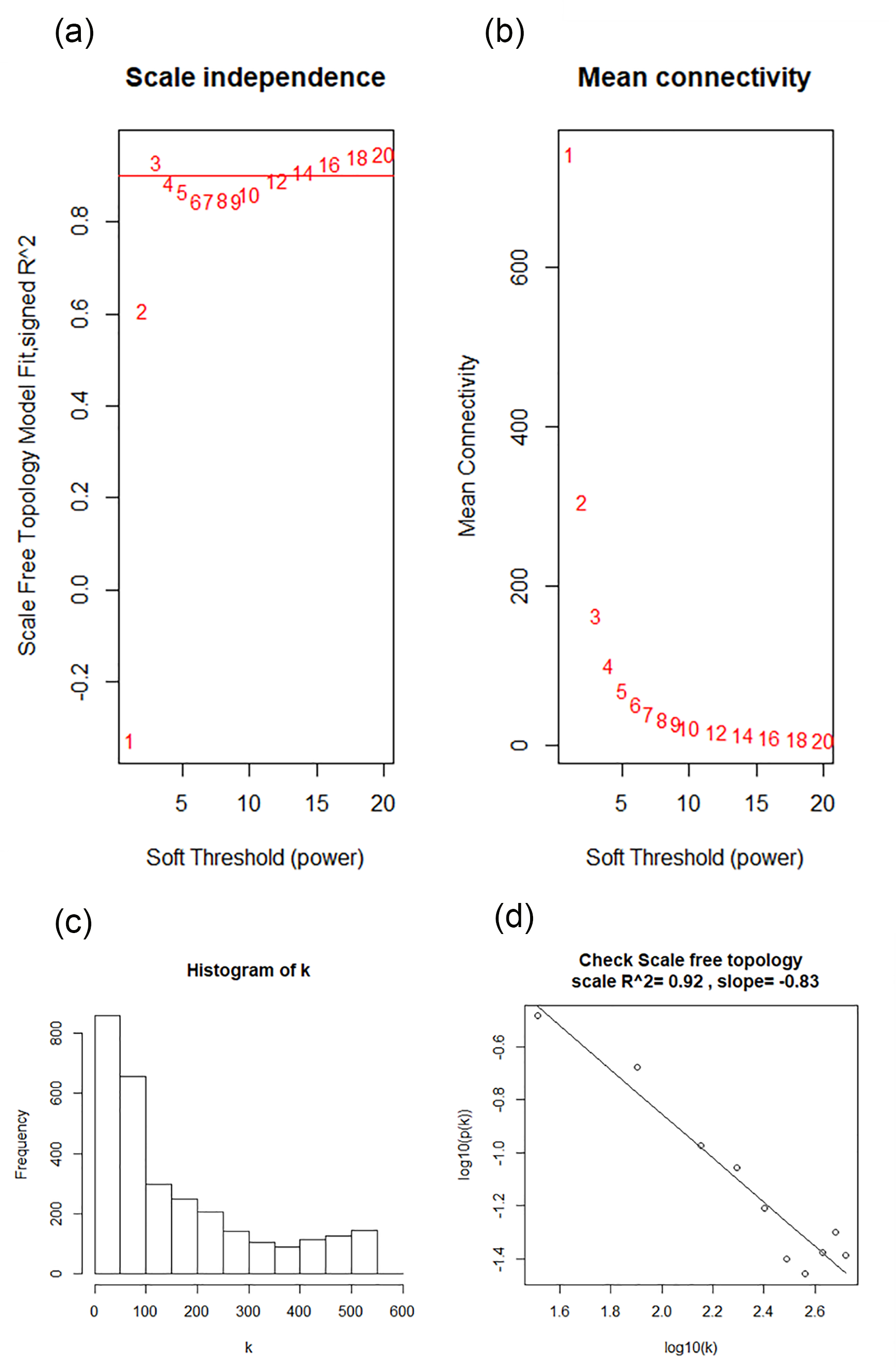

Supplement: FIGURE S2 — Determination of soft-thresholding power in the weighted gene co-expression network analysis (WGCNA). (a) Analysis of the scale-free fit index for various soft-thresholding powers. (b) Analysis of the mean connectivity for various soft-thresholding powers. (c) Histogram of connectivity distribution when β = 3. (d) Checking the scale free topology when β = 3. [file Image_2.TIF]

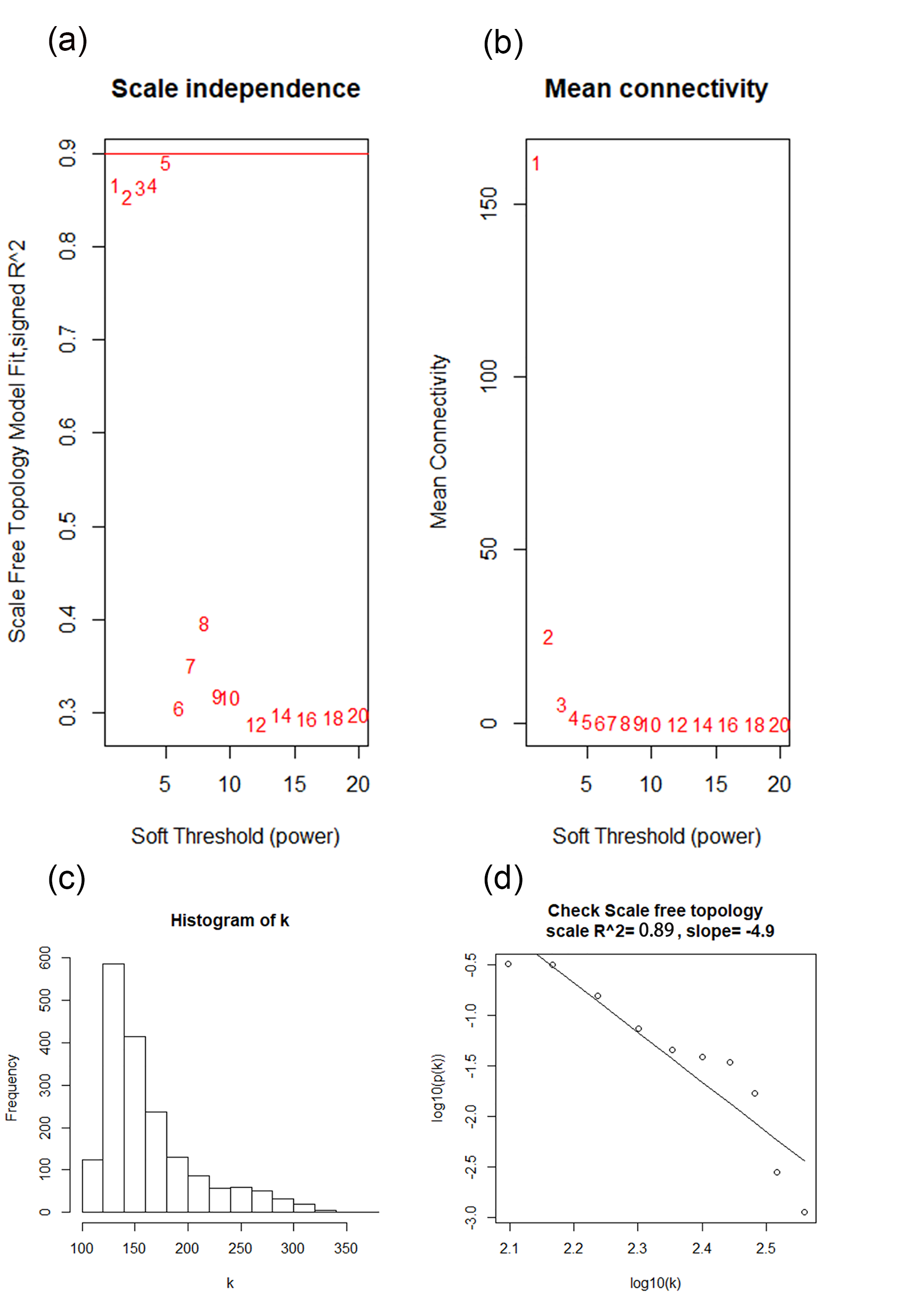

Supplement: FIGURE S3 — Determination of soft-thresholding power in the weighted miRNA co-expression network analysis. (a) Analysis of the scale-free fit index for various soft-thresholding powers. (b) Analysis of the mean connectivity for various soft-thresholding powers. (c) Histogram of connectivity distribution when β = 5. (d) Checking the scale free topology when β = 5. [file Image_3.TIF]

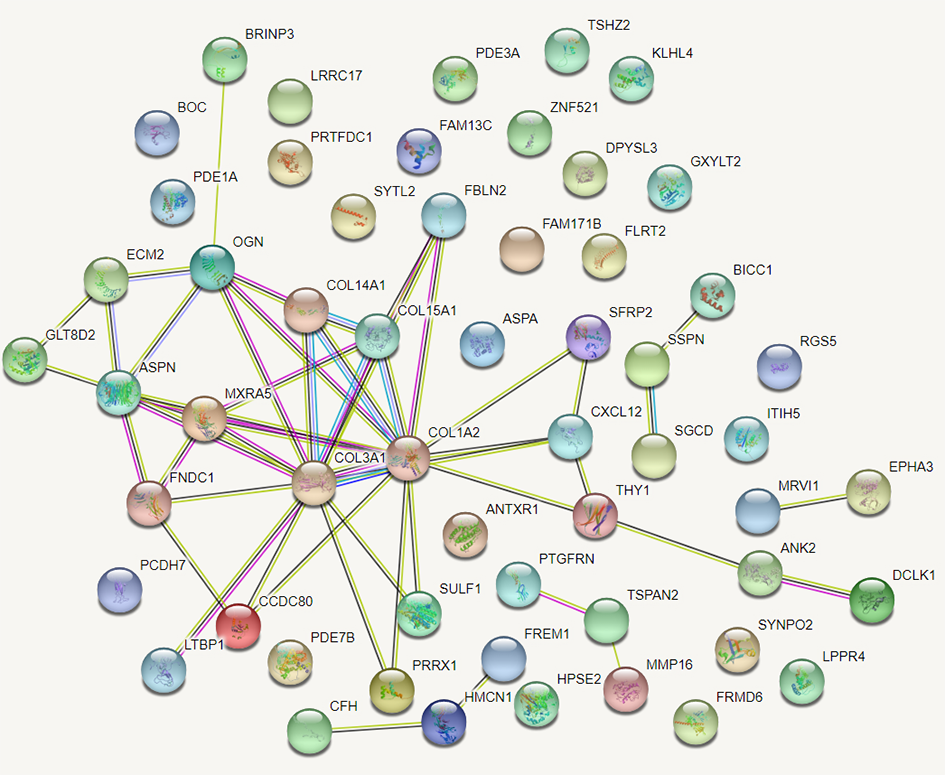

Supplement: FIGURE S4 — Protein–protein interaction network of 58 candidate genes acquired from STRING 9.1. [file Image_4.TIF]

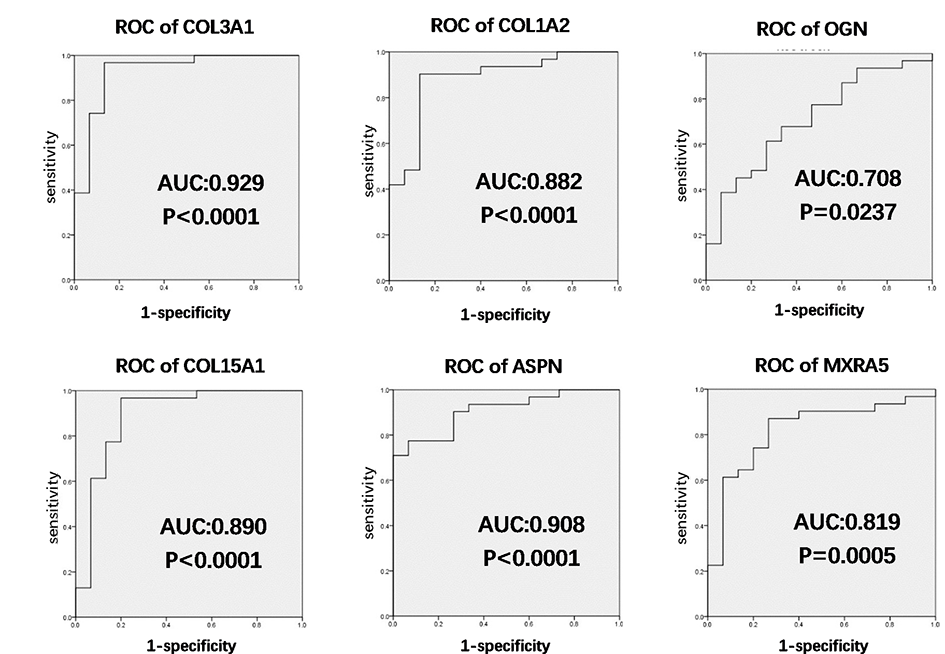

Supplement: FIGURE S5 — ROC curve of COL3A1, COL1A2, OGN, COL15A1, ASPN, and MXRA5 in GSE10067. [file Image_5.TIF]

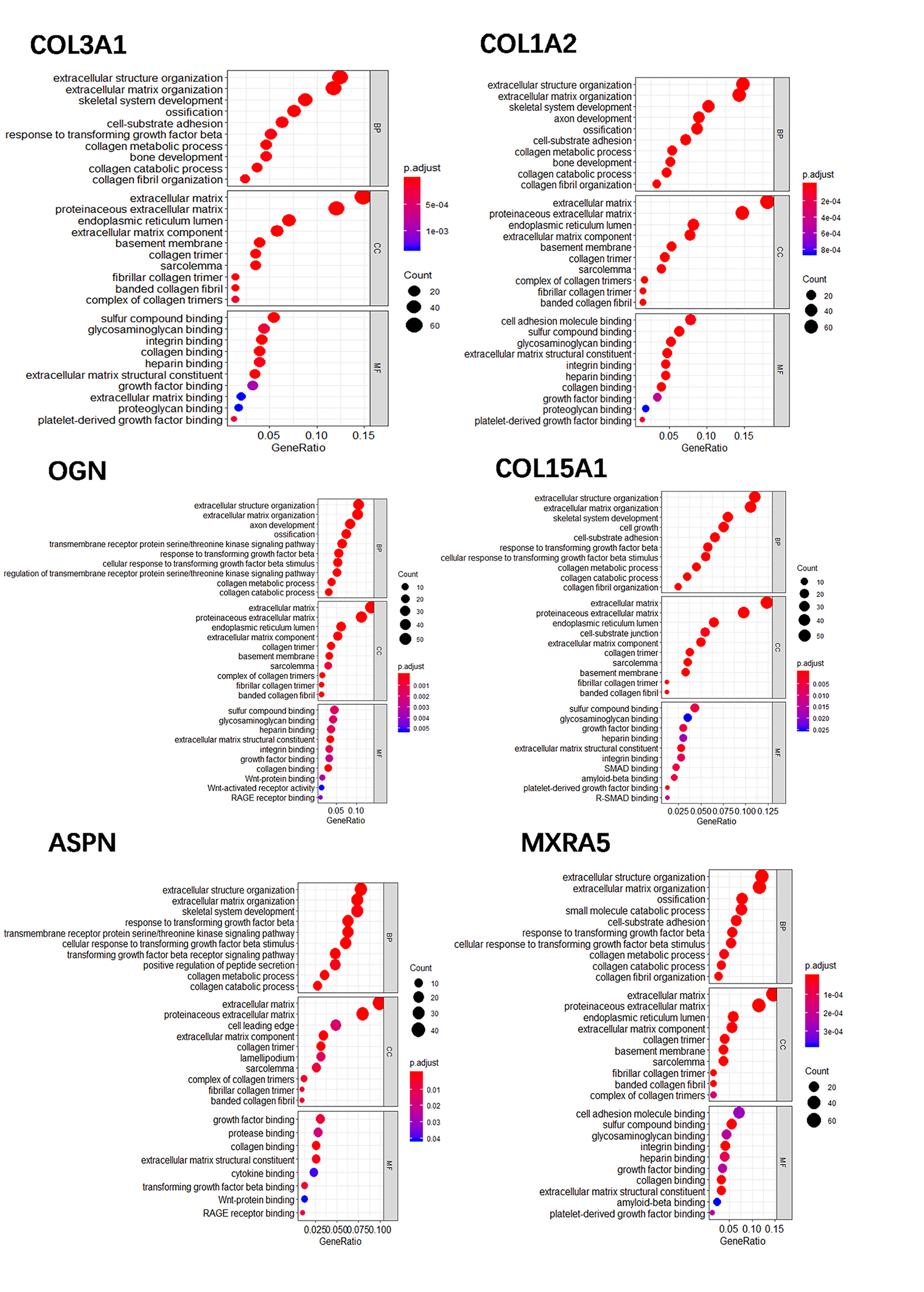

Supplement: FIGURE S6 — Guilt of association for hub genes (COL3A1, COL1A2, OGN, COL15A1, ASPN, and MXRA5). [file Image_6.TIF]
