# Supplementary material for: Analysis of the Interaction Network of Hub miRNAs-Hub Genes, Being Involved in Idiopathic Pulmonary Fibers and Its Emerging Role in Non-small Cell Lung Cancer
Source: Front Genet. 2020 Apr 2;11:302. doi: 10.3389/fgene.2020.00302 (PMC7142269; doi:10.3389/fgene.2020.00302)
Supplement: TABLE S1 — Gene and miRNA expression microarray datasets related to IPF. [file Table_1.DOCX]

**Table1:** **Gene and miRNA expression microarray datasets related to IPF.**

|  | Accession number of the dataset | Platform | Organism | Disease type | |
| --- | --- | --- | --- | --- | --- |
|  |  |  |  | Control | IPF |
| genes | GSE32537 | GPL6244 | Homo sapiens | 50 | 119 |
|  | GSE10667 | GPL4133 | Homo sapiens | 15 | 31 |
|  | GSE70866 | GPL14550 | Homo sapiens | 20 | 110 |
| miRNAs | GSE32538 | GPL8786 | Homo sapiens | 50 | 106 |
|  | GSE27430 | GPL8227 | Homo sapiens | 12 | 13 |

Note. miRNA: microRNA. IPF: idiopathic pulmonary fibers.
