# Supplementary material for: Analysis of the Interaction Network of Hub miRNAs-Hub Genes, Being Involved in Idiopathic Pulmonary Fibers and Its Emerging Role in Non-small Cell Lung Cancer
Source: Front Genet. 2020 Apr 2;11:302. doi: 10.3389/fgene.2020.00302 (PMC7142269; doi:10.3389/fgene.2020.00302)
Supplement: TABLE S2 — Gene set enriched in lung samples with COL3A1 high expression. [file Table_2.DOCX]

**Table S2**: **Gene set enriched in lung samples with COL3A1 high expression.**

| COL3A1 | ES | NES | NOM p-val | FDR q-val |
| --- | --- | --- | --- | --- |
| Cellular Adhesion molecules | 0.536342 | 1.654729 | 0.013015 | 0.053926 |
| ECM receptor interaction | 0.640283 | 1.71831 | 0.013834 | 0.101429 |
| p53_signaling_pathway | 0.606931 | 1.654092 | 0.014141 | 0.189836 |
| Taurine and hypo taurine metabolism | 0.78284 | 1.530532 | 0.016097 | 0.049822 |
| Focal adhesion | 0.517178 | 1.710034 | 0.018443 | 0.205226 |
| Melanoma | 0.513445 | 1.609675 | 0.020534 | 0.197992 |
| Nicotinate and nicotinamide metabolism | 0.706595 | 1.53601 | 0.026369 | 0.225626 |
| Cell cycle | 0.575696 | 1.592027 | 0.033473 | 0.081134 |
| Alanine aspartate and glutamate metabolism | 0.555106 | 1.507612 | 0.039448 | 0.244856 |
| Arrhythmogenic right ventricular cardiomyopathy ARVC | 0.525595 | 1.532511 | 0.044266 | 0.246926 |

Note. ES, enrichment score; NES, normalized enrichment score; NOM p-val, nominal p value; FDR, false discovery rate q value. ECM, extracellular matrix. ARVC, arrhythmogenic right ventricular cardiomyopathy.
