# Supplementary material for: Analysis of the Interaction Network of Hub miRNAs-Hub Genes, Being Involved in Idiopathic Pulmonary Fibers and Its Emerging Role in Non-small Cell Lung Cancer
Source: Front Genet. 2020 Apr 2;11:302. doi: 10.3389/fgene.2020.00302 (PMC7142269; doi:10.3389/fgene.2020.00302)
Supplement: TABLE S3 — Gene set enriched in lung samples with COL1A2 high expression. [file Table_3.DOCX]

**Table S3**: **Gene set enriched in lung samples with COL1A2 high expression.**

| COL1A2 | ES | NES | NOM p-val | FDR q-val |
| --- | --- | --- | --- | --- |
| ECM receptor interaction | 0.634084 | 1.886428 | 0.001949 | 0.085017 |
| Focal adhesion | 0.502074 | 1.778817 | 0.013725 | 0.086826 |
| Cell adhesion molecules cams | 0.552271 | 1.660345 | 0.017787 | 0.1576 |
| Dilated cardiomyopathy | 0.507821 | 1.588004 | 0.045187 | 0.192992 |
| Arrhythmogenic right ventricular cardiomyopathy ARVC | 0.52528 | 1.567251 | 0.035857 | 0.196048 |

Note. ES, enrichment score; NES, normalized enrichment score; NOM p-val, nominal p value; FDR, false discovery rate q value. ECM, extracellular matrix. ARVC, arrhythmogenic right ventricular cardiomyopathy.
