# Supplementary material for: Analysis of the Interaction Network of Hub miRNAs-Hub Genes, Being Involved in Idiopathic Pulmonary Fibers and Its Emerging Role in Non-small Cell Lung Cancer
Source: Front Genet. 2020 Apr 2;11:302. doi: 10.3389/fgene.2020.00302 (PMC7142269; doi:10.3389/fgene.2020.00302)
Supplement: TABLE S4 — Gene set enriched in lung samples with OGN high expression. [file Table_4.DOCX]

**Table S4**: **Gene set enriched in lung samples with OGN high expression.**

| OGN | ES | NES | NOM p-val | FDR q-val |
| --- | --- | --- | --- | --- |
| ECM receptor interaction | 0.535628 | 1.580543 | 0.04 | 0.098781 |
| Arrhythmogenic right ventricular cardiomyopathy ARVC | 0.504738 | 1.503061 | 0.045908 | 0.156042 |
| Taurine and hypo taurine metabolism | 0.737833 | 1.469451 | 0.046843 | 0.141391 |
| Histidine metabolism | 0.539988 | 1.463656 | 0.045369 | 0.163312 |

Note. ES, enrichment score; NES, normalized enrichment score; NOM p-val, nominal p value; FDR, false discovery rate q value. ECM, extracellular matrix. ARVC, arrhythmogenic right ventricular cardiomyopathy.
