# Supplementary material for: Analysis of the Interaction Network of Hub miRNAs-Hub Genes, Being Involved in Idiopathic Pulmonary Fibers and Its Emerging Role in Non-small Cell Lung Cancer
Source: Front Genet. 2020 Apr 2;11:302. doi: 10.3389/fgene.2020.00302 (PMC7142269; doi:10.3389/fgene.2020.00302)
Supplement: TABLE S5 — Gene set enriched in lung samples with COL15A1 high expression. [file Table_5.DOCX]

**Table S5**: **Gene set enriched in lung samples with COL15A1 high expression.**

| COL15A1 | ES | NES | NOM p-val | FDR q-val |
| --- | --- | --- | --- | --- |
| ECM receptor interaction | 0.537338 | 1.55207 | 0.014099 | 0.015361 |
| Histidine metabolism | 0.539804 | 1.463656 | 0.014909 | 0.023611 |
| Nicotinate and nicotinamide metabolism | 0.59753 | 1.326687 | 0.021984 | 0.048747 |
| Glycosaminoglycan biosynthesis keratan sulfate | 0.51991 | 1.311151 | 0.026371 | 0.053782 |
| Mismatch repair | 0.523668 | 1.287296 | 0.028819 | 0.091032 |
| Homologous recombination | 0.523511 | 1.247955 | 0.035427 | 0.090087 |
| DNA replication | 0.514823 | 1.246602 | 0.036925 | 0.152033 |
| Asthma | 0.532948 | 1.198557 | 0.039718 | 0.192256 |
| Ascorbate and aldarate metabolism | 0.593589 | 1.152484 | 0.040458 | 0.207375 |
| Porphyrin and chlorophyll metabolism | 0.507171 | 1.100106 | 0.041213 | 0.218461 |
| Taurine and hypo taurine metabolism | 0.525317 | 1.059512 | 0.044694 | 0.220138 |

Note. ES, enrichment score; NES, normalized enrichment score; NOM p-val, nominal p value; FDR, false discovery rate q value. ECM, extracellular matrix.
