# Supplementary material for: Analysis of the Interaction Network of Hub miRNAs-Hub Genes, Being Involved in Idiopathic Pulmonary Fibers and Its Emerging Role in Non-small Cell Lung Cancer
Source: Front Genet. 2020 Apr 2;11:302. doi: 10.3389/fgene.2020.00302 (PMC7142269; doi:10.3389/fgene.2020.00302)
Supplement: TABLE S6 — Gene set enriched in lung samples with ASPN high expression. [file Table_6.DOCX]

**Table S6**: Gene set enriched in lung samples with ASPN high expression.

| ASPN | ES | NES | NOM p-val | FDR q-val |
| --- | --- | --- | --- | --- |
| WNT signaling pathway | 0.513819 | 1.527173 | 0.010404 | 0.118729 |
| Purine metabolism | 0.521802 | 1.448414 | 0.015082 | 0.155176 |
| Histidine metabolism | 0.528343 | 1.423969 | 0.027369 | 0.168541 |
| Homologous recombination | 0.578335 | 1.365947 | 0.034503 | 0.185935 |
| ECM receptor interaction | 0.654031 | 1.341087 | 0.044329 | 0.192542 |
| Progesterone mediated oocyte maturation | 0.592901 | 1.331644 | 0.045039 | 0.195328 |
| Taurine and hypo taurine metabolism | 0.649025 | 1.299528 | 0.046129 | 0.159281 |

Note. ES, enrichment score; NES, normalized enrichment score; NOM p-val, nominal p value; FDR, false discovery rate q value. ECM, extracellular matrix.
