# Supplementary material for: Analysis of the Interaction Network of Hub miRNAs-Hub Genes, Being Involved in Idiopathic Pulmonary Fibers and Its Emerging Role in Non-small Cell Lung Cancer
Source: Front Genet. 2020 Apr 2;11:302. doi: 10.3389/fgene.2020.00302 (PMC7142269; doi:10.3389/fgene.2020.00302)
Supplement: TABLE S7 — Gene set enriched in lung samples with MXRA5 high expression. [file Table_7.DOCX]

**Table S7**: Gene set enriched in lung samples with MXRA5 high expression.

| MXRA5 | ES | NES | NOM p-val | FDR q-val |
| --- | --- | --- | --- | --- |
| ECM receptor interaction | 0.575357 | 1.714133 | 0.017316 | 0.066866 |
| p53 signaling pathway | 0.520337 | 1.547521 | 0.019671 | 0.075625 |
| Histidine metabolism | 0.535164 | 1.473161 | 0.022738 | 0.082408 |
| Nicotinate and nicotinamide metabolism | 0.644138 | 1.446821 | 0.022909 | 0.059589 |
| Intestinal immune network for IGA production | 0.577054 | 1.40149 | 0.025246 | 0.125644 |
| Asthma | 0.593154 | 1.315028 | 0.027068 | 0.154271 |
| Glycosaminoglycan biosynthesis heparan sulfate | 0.504454 | 1.30558 | 0.028992 | 0.168431 |
| Taurine and hypo taurine metabolism | 0.629073 | 1.271103 | 0.031235 | 0.180835 |
| Primary bile acid biosynthesis | 0.580023 | 1.241478 | 0.035218 | 0.184796 |
| Primary immunodeficiency | 0.559964 | 1.221829 | 0.036601 | 0.199438 |
| Ascorbate and aldarate metabolism | 0.615405 | 1.182156 | 0.041157 | 0.209024 |
| Porphyrin and chlorophyll metabolism | 0.53866 | 1.146449 | 0.049243 | 0.215151 |

Note. ES, enrichment score; NES, normalized enrichment score; NOM p-val, nominal p value; FDR, false discovery rate q value. ECM, extracellular matrix.
