# Supplementary material for: Analysis of the Interaction Network of Hub miRNAs-Hub Genes, Being Involved in Idiopathic Pulmonary Fibers and Its Emerging Role in Non-small Cell Lung Cancer
Source: Front Genet. 2020 Apr 2;11:302. doi: 10.3389/fgene.2020.00302 (PMC7142269; doi:10.3389/fgene.2020.00302)
Supplement: TABLE S8 — Clinicopathological correlation analysis for hub genes and hub miRNAs in IPF. [file Table_8.DOCX]

**Table S8:** **Clinicopathological correlation analysis for hub genes and hub miRNAs in IPF.**

|  | **Symbol** | **p-Value (age)** | **p-Value (gender)** |
| --- | --- | --- | --- |
| **Hub Genes** | COL3A1 | 0.126351 | 0.057855 |
|  | COL1A2 | 0.656548 | 0.112127 |
|  | OGN | 0.362548 | 0.216074 |
|  | COL15A1 | 0.035691 | 0.600289 |
|  | ASPN | 0.053694 | 0.156792 |
|  | MXRA5 | 0.153368 | 0.026355 |
| **Hub miRNAs** | hsa-let-7b-5p | 0.398847 | 0.032564 |
|  | hsa-miR-26a-5p | 0.856921 | 0.632158 |
|  | hsa-miR-25-3p | 0.042311 | 0.326498 |
|  | hsa-miR-29c-3p | 0.693124 | 0.045612 |
|  | hsa-let-7c-5p | 0.083604 | 0.065312 |
|  | hsa-miR-29b-3p | 0.096341 | 0.073641 |
|  | hsa-miR-26b-5p | 0.536915 | 0.262147 |

Note. miRNA: microRNA.
